# The Effect of Prenatal Exposure to Restraint Stress on Hippocampal Granule Neurons of Adult Rat Offspring

**Published:** 2012

**Authors:** Mohammad Hosseini-sharifabad, Ebrahim Esfandiari, Ali Hosseini-sharifabad

**Affiliations:** 1 *Department of Cell Biology and Anatomy, Shahid Sadoughi University of Medical Sciences, Yazd, Iran*; 2 *Department of Biomedical Sciences, Isfahan University of Medical Sciences, Isfahan, Iran*; 3 *Department of Pharmacology and Toxicology, Pharmacy Faculty, Shahid Sadoughi University of Medical Sciences, yazd, Iran*

**Keywords:** Dentate gyrus, Hippocampus, Neuron, Number, Prenatal stress, Volume

## Abstract

**Objective(s):**

It is well known that prenatal stresses (PS) induce a variety of neurobiological and behavioral alterations, some of them involving the hippocampal formation. This study aimed to determine whether restraint stress influences the neuronal volume and number of granule cells in the hippocampus of adult rat offspring.

**Materials and Methods:**

Ten Wistar pregnant rats were randomly divided: stressed and control groups. Pregnant dams in the stressed group were placed in a Plexiglas restraint tube for 1 hr daily from days 15-21 of gestation. Neuroendocrinological consequences of prenatal stress exposure were evaluated in the male offspring on postnatal day 60. The total numbers and the individual volume of granule cells in the hippocampus were also estimated with the optical fractionator and the rotator methods, respectively.

**Results:**

Prenatally stressed rats exhibited prolonged elevation in plasma glucocorticoid levels following acute exposure to restraint stress. Data also indicated that there is a decrease in neuronal volume of hippocampal granule cells in prenatally stressed compared with their controls (625±64.1 µm^3^ vs. 741±80.6 µm^3^). There was no significant difference in the total number of granule cells between prenatally stressed and control animals.

**Conclusion:**

The present study indicated that exposure of pregnant female during last week of pregnancy leads to a decline in neuronal size in hippocampus of adult male rats without neuronal loss. The present results may provide a basis for the understanding of the reported disturbances in behavior and learning of PS offspring.

## Introduction

It is well known that prenatal stress (PS) induces long-lasting neurobilogical and behavioural alterations in adult offspring (1, 2).

Accumulating literature reported that PS disturbs the regulation of hypothalamo-pituitary-adrenal (HPA) axis. As a consequence, PS offspring cannot react appropriately to stressful life events (3-6) and this may lead to permanent sensitization of the brain to stressful situations. A number of studies focused on the hippocampus to elucidate the molecular and cellular mechanisms by which early-life stress induces long-term changes and plasticity in the CNS.

It is well established that the development of the dentate gyrus of hippocampus begins during gestation and continue to postnatal period. (7, 8). 

Several studies have suggested that PS inhibits neurogenesis of dentate granule cells (9- 14), but only a few studies have examined the effect of PS on the neuronal number of dentate gyrus in adult offspring (13, 15, 16). For example, lemaire *et al* (2000) reported that PS inhibits cell proliferation in the dentate gyrus of adult rats. In addition, they showed that the dentate gyrus of both juvenile and adult male PS rats had fewer neurons than non-stressed controls (13).

Other investigators have reported a reduced number of granule cells in the hippocampus of adult female rats but not males due to prenatal restraint stress (15, 16)**.**

Because prenatal stress reduces cell proliferation in the dentate gyrus, it is of interest to evaluate exactly the consequences of prenatal stress on the morphometerical aspects of granule neurons.

We previously examined the effect of PS on the size of hippocampus in male rats. The result showed that two months old male offspring exposed to restraint stress in the last week of gestation had remarkable size decrease in the dentate gyrus subregion of hippocampus (17). Reported decrease in hippocampal size might be due to a reduction in the size of constituent neurons or as a result of neuronal loss. Therefore, in continuing the previous works, we examined the influence of PS on the total number of neuron and, to our knowledge, for the first time, the individual neuronal volume in the hippocampus of male adult rats.

Since advances in the field of stereology provide the possibility for a very precise estimation of the number and size of neurons; we employed design-based stereological methods (18) to quantify changes occurring in the dentate gyrus, following exposure to PS. 

## Materials and Methods

***Animals***

Ten virgin female Wistar rats weighing 230-250 g (procured from animal house of Isfahan Medical Faculty, Iran) were housed in the presence of a sexually experienced Wistar male weighing 450-500 g. Vaginal smears were obtained each morning to detect mating. The pregnant animals were individually housed with free access to rat feeds (Khorak Daam Pars, Iran) and water in a temperature-controlled (22±2 °C) animal room, on a 12 hr light/dark cycle (light on: 07.00-19.00 hr; light off: 19.00-07.00 hr).

The pregnant dams were assigned randomly to control and study groups. Animal care and handling was performed in accordance with rules approved by the local research council of Shahid Sadoughi Medical University of Iran.

*Induction of prenatal stress*

During the last week of pregnancy, from day 15 until delivery, pregnant females in the study group were individually restrained for 1 hr a day (08.00-09.00 hr). This stress procedure was described by Ward and Weisz (1984) and was chosen because it has an indirect influence on the fetus via direct stress on the mother (19). The restraint device was a transparent plastic tube (7 cm in diameter) with air holes for breathing and a closed end. The length could be adjusted to accommodate the size of the animal. Rats in the control group were undisturbed in their home cages.

All dams delivered their offspring vaginally. In each group, only 8-12 similar sex offspring were studied. On day 21, after all offspring were weaned, female and male offspring were separated and housed, four in each cage. A maximum of two male offspring were taken from each litter to remove any ‘litter effects’ (20). The animals were exposed to normal animal room conditions until testing at two months of age.

*Corticosterone assay*

Corticosterone levels of rat offspring were measured in blood samples drawn from the tail vein on three occasions: before stress, after 20 min restraint, and 120 min after restraint between 09.00 and 12.00 hr. The rats were restrained in plastic cylinders identical to those used for the prenatal stress procedure. Blood corticosterone levels were determined by radioimmunoassay using kits purchased from ICN Pharmaceuticals (). Assays were conducted according to the protocols provided by the manufacturer.

*Histological procedure*

Rats were deeply anesthetized with urethan (Merk, Germany) and transcardially perfused with a phosphate-buffered solution (pH 7.2, 0.12 mol/l) of 4% formaldehyde and 1% glutaraldehyde. The brains were removed, numbered, and the cerebellum and olfactory bulbs were removed. Brains were weighed and the cerebral hemispheres were separated by a longitudinal cut in midsagital plane. Each was placed in a chilled slicing box and the first frontal of the cerebral hemispheres were removed and the following , which contained hippocampus, were collected. One hemisphere was selected at random for estimating number of neurons and the other for estimating volume of individual neurons. Stereologic analyses were performed at the Stereology and Electron Microscopy Research Laboratory, Centre for Stochastic Geometry and Advanced Bioimaging, University of Aarhus, Aarhus, Denmark.

***Estimation of neuron number***

Coronal sections of 100 µm thickness were cut serially, with a calibrated vibratome (Bio-Rad Polaron H1200, UK), and were collected along the entire extent of the hippocampus. One of the five series of sections was selected for staining, using systematic uniformly random sampling. The sections were stained free floating in hematoxylin, dehydrated, and immersed in Epon resin. The sections were then transferred onto slides and coverslipped. They were dried overnight at 60 C for polymerizing (21).

Stereological analyses were carried out under blind conditions with an optical fractionator with varying sampling fractions, which is suited for use with shrunken vibratome sections (18).

The initial delineation of the dentate gyrus was performed using a 4x objective (Olympus, Splan, NA 0.16) at a final magnification of 157x. 

**Figure 1:**
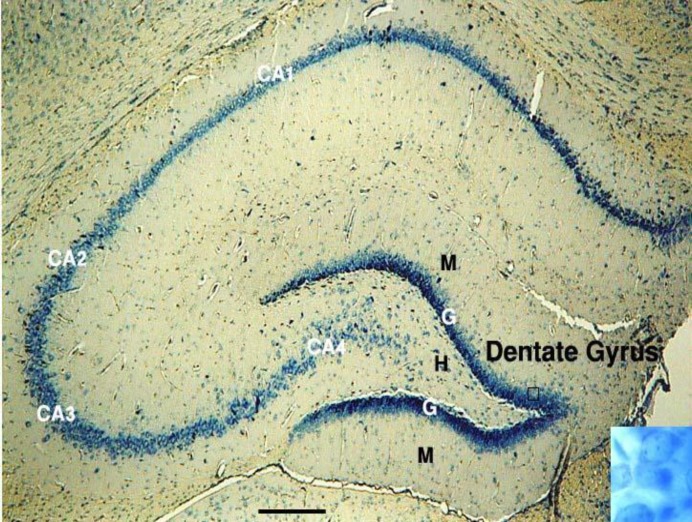
Light micrograph of a section through the rat hippocampus (Giemsa stain, scale bar=500 µm). Three layers of dentate gyrus: Molecular (M), Granule cell (G), and Hilus (H). The CA1, CA2, CA3, and CA4 are subfields of Cornu Ammonis (CA). Higher magnification (1730x) of dentate granule cells are shown in the lower box

Dentate Gyrus is an easily recognized region due to intense staining and because it is not continuous with the other hippocampal regions (Figure1). 

The sections were analyzed using a 60x oil immersion objective (Olympus, Splan, NA 1.40) at a final magnification of 2373x. Glial cells, characterized by their much smaller size compared with the neighboring neurons as well as peculiar cytological features (dense bodies and large nuclei surrounded by a sparse cytoplasm) (22) were not included in the estimates. The basket cells which comprise less than 1% of the neurons of these layers (23) were not easily distinguishable from the surrounding neurons and were therefore included in the estimates.

All quantifications were done with the CAST software, version 2.1 (Visiopharm, Hørsholm, Denmark). Sampling schemes for optical disectors used to estimate the number of granule cells in the dentate gyrus of hippocampus is summarized in Table 1.

***Estimation of neuron volume***

Each brain block containing hippocampus was sectioned coronally on the vibratome into a thin section (100 µm) and a thick slice (1.5 mm), alternately. The thin sections were mounted onto a gelatinized glass slide and stained with hematoxylin and were used for identification of the exact position within the hippocampus. 

The thick slices (1.5 mm) were used for “vertical sections” (24). Using a section plane parallel to the chosen axis but uniformly randomly rotated, the slabs were cut in bars with 1 mm width. Each bar containing hippocampi was turned 90  around the long (vertical) axis so that histological sections from the vertical edge of a bar could be produced. The bars from each slice were then embedded in 5% agar. In order to quantify shrinkage caused by fixation and histological procedures, measurements were made before and after processing. The bars were dehydrated by a graded series of ethanol solutions, 70% (2 5 hr), 96% (2 2 hr), and 99% ethanol (3 2 hr) and infiltrated with glycol methacrylate (Technovit 7100, Kulzer, Germany) changed daily for 6 days. The bars were embedded at room temperature and the blocks were ready for sectioning after (about) 24 hr.

Sections were cut with HM 355 rotary microtome (MICROM International GMBH, Walldorf, Germany) using glass knives with a microtome setting of 40  m. The sections were mounted onto ordinary glass slides, and immediately dried at 60  C prior to staining.

The sections were stained with a Giemsa stain (25) modified for use with glycol methacrylate embedded tissue. The staining solution contained 10 ml Giemsa stain stock solution (KEBO Lab A/S, Copenhagen, product 50436-1), 40 ml distilled water, and 2 drops of 1% acetic acid. The mounted sections were placed in the staining solution for 70 min at room temperature, rinsed in 1% acetic acid for 3 min, and differentiated in 96% ethanol for 30 min and again in 99% ethanol for 30 min. The sections were dried without coverslips.

Since shrinkage influences all stereological size estimators, including volume, measurements were made to quantify shrinkage caused by fixation and histological procedures.

Sampling schemes for optical disectors used to the neuron sampling process is summarized in Table 1. The volume measurement was performed by the use of the vertical rotator (26) implemented in CAST software. 

***Statistical analysis***

The results were then subjected to Student's t-test, with significance determined at the *P*< 0.05. The coefficient of error (CE) of the number estimates was calculated according to Gundersen *et al* (27). The coefficient of variation (CV) is defined as CV=SD/mean.

**Table 1:** Sampling schemes for optical disectors used to estimate the number and the individual volume of granule cells in the dentate gyrus of hippocampus. Distance between disectors in orthogonal directions x and y on the sections are dx and dy

	Number	Volume
Frame area	138 m^2^	138 m^2^
dx . dy	180×180 m	70×70 m
Disector height	15 m	15 m
Upper guard zone	10 μm	10 μm

## Results

The brain weight of PS rats did not show a significant difference but the body weight of stressed animals were significantly lower than those in control animals (Table 2)

**Figure 2:**
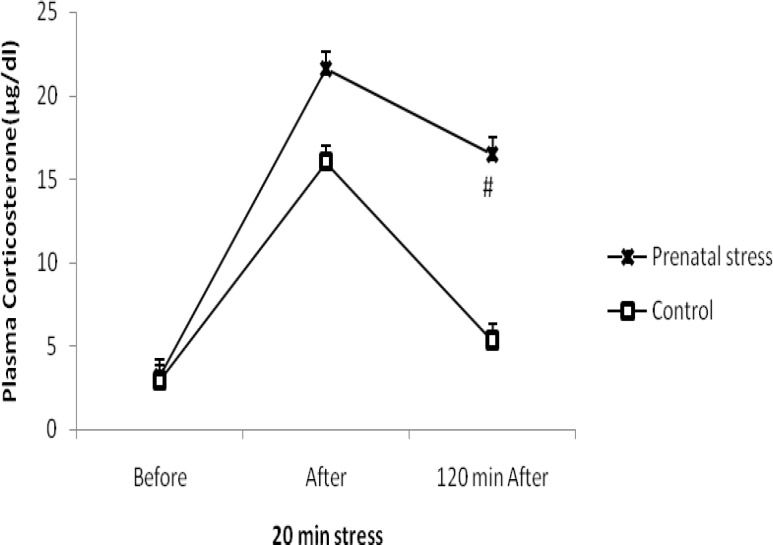
Effect of a 20 min exposure to restraint stress on plasma corticosterone concentrations in PS rats and controls. Values are expressed as mean±SD (8 rats in each group, *P*< 0.001)

Plasma corticosterone levels, measured immediately before and after and 120 min after the 20 min restraint stress, is shown in Figure 2. A prolonged elevation in plasma corticosterone was observed in adult rats exposed to stress during the third week of gestation which was significantly greater than baseline values at the 120 min time point.

In control rats, exposure to a 20 min period of restraint elicits significant increase in corticosterone levels observed at the end of the 20 min stress and a return to baseline levels 120 min after termination of the stressor (*P*> 0.05, Figure 2). 

Furthermore, prenatal stress had no effect on basal plasma corticostrone level (i.e., before stress, *P*> 0.05).

Using sampling parameters in stereological analyses (Table 1), 117 optical disectors were examined in granule cell layer and the mean of 167 cells were counted per hippocampus.

As shown in Table 3, no significant effect of PS was found on the total number of neurons in the dentate granule cells of hippocampus. The means of CEs of the number estimates ranged from 0.07 to 0.09.

Results also showed that there is a significant effect of PS on decreasing the individual volume of the dentate granule cells (Table 3).

Due to the significant magnitude of volume shrinkage (on average 20%) of the glycol methacrylate embedded sections, the individual neuron volumes in Table 3 were corrected for tissue shrinkage.

**Figure 3:**
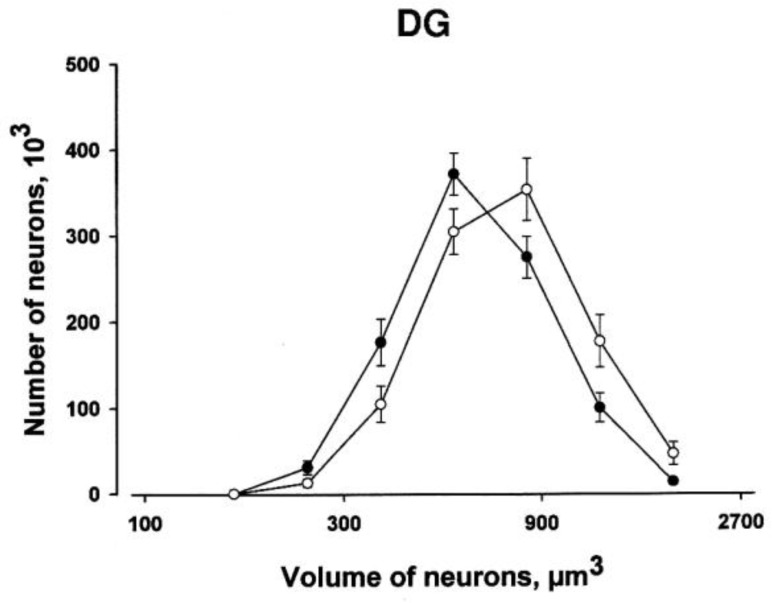
The absolute distribution of somal volume estimates for dentate granular layer of hippocampus. The filled circle indicates the mean distribution of the prenatally stressed (PS) group while the empty circle represents the control group. Vertical bars represent SEM. The difference in somal volume is easily seen as a left shift of the estimated size distribution in controls compared with PS group. Each of the two graphs is based on approximately 2500 individual somal volume estimates

Figure 3 shows a substantial skewing of the distributions toward smaller somal sizes of dentate granule neurons in prenatally stressed rats.

**Table 2:** Body and brain weights in control and prenatally stressed (PS) rat

	Control	PS
Body weight	205.9±12.8	179.6±21.9^*^
Brain weight	1.33± 0.09	1.29±0.08 NS

Value represent the mean weight (g)±SD, ^*^ *P*<0.05

**Table 3:** Means of number (N) and individual volume (µm^3^) of dentate granule cells of prenatally stressed (PS) and respective controls. The coefficient of variance CV=SD/mean in the parentheses. CE stands for the coefficient of error

Groups	Dentate granule cells		
	N	CE (N)	Cell volume (µm^3^)
Control (n=8)	0.999×106 (0.13)	0.08	741 (0.11)
PS (n=9)	0.967×106 (0.16) *P*= 0.86	0.08	625 (0.10) *P*= 0.005

## Discussion

The main findings of this study are as follow: 1) exposure of pregnant female rats to restraint stress during the third week of pregnancy results in a prolongation of corticosterone response to an acute stress in adult offspring. 2) Prenatal stress induces decreased neuronal size in granule cells of dentate gyrus without neuronal loss.

Numerous studies revealed that adult PS rats exhibit changes in the HPA axis activity. However, the reported pattern of alteration is not always consistent. The most commonly found change in HPA axis activity, consistent with our results, is a prolongation of glucocorticoid response to an acute stress in the adult PS offspring (5, 6, 28, 29) but some studies also found a shift in basal levels of HPA axis activity (30, 31).

Previous studies showed that the prolongation of the acute stress response in PS rat is most likely as a consequence of a decrease in the expression of glucocorticoid receptors in the hippocampus (5, 6**)**, although we did not measure glucocorticoid receptor expression in our experiments. It is also shown that PS increases maternal corticosterone (19, 28) which readily crosses placental and blood-brain barriers and entering into the fetal circulatory system and brain (32).

Various prenatal stresses have been reported to induce structural alterations in the hippocampal formation. It is well known that relatively severe forms of prenatal stress such as prolonged noise, repeated daily restraint, and repeated saline injections can cause decreased neurogenesis in the dentate gyrus of hippocampus (9-14).

Our results in adult Wistar rats are consistent with the view that PS does not lead to significant cell loss in any of the main populations of hippocampal formation of adult male offspring (15, 16). However, they differ from another report claiming that PS results in neuronal loss in the dentate gyrus of the hippocampus in adult male rats (13). The reasons for these discrepancies are not readily apparent but may be due to the intensity and duration of the maternal stress, the age of the rats when tested, and how the neuronal number were measured.

However, it is important to note that the glucocorticoid effects upon the structure of the dentate gyrus may not merely be manifested as changes in total cell numbers.

In order to search for other prenatal stress-induced structural changes at the light microscopic level, we have also evaluated the individual somal volumes of the dentate granule cells. In our search, there has been no previous attempt to estimate individual volume of hippocampal neurons after exposure to PS using unbiased principles. The present study demonstrated for the first time that PS diminished the individual somal volume of dentate granule cells. Decrease in the perikaryal volume might cause a more global effect due to the decreased contents of neurotransmitters, intracellular organelles, and the number of synaptic contacts. (33-35).

Some studies have shown that stress during pregnancy induces morphological changes in processes of hippocampal neurons in the adult offspring (36, 37).

It is noteworthy to mention that the quantitative techniques used in the current study were sufficiently sensitive to detect even small PS-related changes in neuronal number and volume. Methods for calculating coefficients of error provide means for evaluating the precision of neuron number and volume estimates derived by stereology (27, 38).

These explanations can be given for the observed atrophy of dentate granule neurons: Prenatal stress prolongs the glucocorticoid secretion following exposure to acute stress in adult offspring (5, 6, 28, 29). Circulating glucocorticoids involve in the cellular mechanism that produces neuronal atrophy in hippocampus (39-41). 

Previous studies also demonstrated that glucocorticoids are regulators of brain neurotrophin levels and an increase of glucocorticoids could reduce mRNA levels for neurotrophins in the hippocampus (42). Neurotrophins influence the physiology and morphology of adult central neurons (43, 44). It is known that a reduction in the neurotrophin level can lead to neuronal atrophy without necessarily being accompanied by neuronal loss (45).

## Conclusions

The present study indicated that exposure of pregnant female rats during last week of pregnancy leads to a decline in neuronal size in hippocampus of adult male rats without neuronal loss. The present results may provide a basis for the understanding of the reported disturbances in behavior and learning of PS offspring.
